# Supplementary material for: Discovery and application of insertion-deletion (INDEL) polymorphisms for QTL mapping of early life-history traits in Atlantic salmon
Source: BMC Genomics. 2010 Mar 8;11:156. doi: 10.1186/1471-2164-11-156 (PMC2838853; doi:10.1186/1471-2164-11-156)
Supplement: Additional file 2 — Information on developed 76 locus single-run INDEL panel in Atlantic salmon. Information on fluorescence labeling, primer concentrations, PCR pooling and links to alignments, INDEL motifs and GENESCAN (Burge and Karlin 1997) predictions of genes/exons are available in html format. [file 1471-2164-11-156-S2.ZIP › Additionalfile2/table3960.html]

Contig Alignment

```
Base	A B C D E F G H I J K   
1	. . . . . . . . C . .    
2	. . . . . . . . G . .    
3	. . . . . . . . C . .    
4	. . . . . . . . T . .    
5	. . . . . . . . G . .    
6	. . . . . . . . G . .    
7	. . . . . . . . G . .    
8	. . . . . . . . G . .    
9	. . . . . . . . A . .    
10	. . . . . . . . G . .    
11	. . . . . . . . A . .    
12	. . . . . . . . T . .    
13	. . . . . . . . A . .    
14	. . . . . . . . G . .    
15	. . . . . . . C C . .    
16	. . . . . . . A A . .    
17	. . . . . . . A A . .    
18	. . . . . . . T T . .    
19	. . . . . . . G G . .    
20	. . . . . . . T T . .    
21	. . . . . . . G G . .    
22	. . . . . . . C C . .    
23	. . . . . . . T T . .    
24	. . . . . . . G G . .    
25	. . . . . . . T T . .    
26	. . . . . . . C C . .    
27	. . . . . . . A A . .    
28	. . . . . . . A A . .    
29	. . . . . . . C C . .    
30	. . . . . . . G G . .    
31	. . . . . . . T T . .    
32	. . . . . . . T T . .    
33	. . . . . . . T T . .    
34	. . . . . . . T T . .    
35	. . . . . . . A A . .    
36	. . . . . . . C C . .    
37	. . . . . . . G G . .    
38	. . . . . . . G G . .    
39	. . . . . . . A A . .    
40	. . . . . . . T T . .    
41	. . . . . . . C C . .    
42	. A . . . . . A A A .    
43	. T . . . . . T T T .    
44	. T . . . . . T T T .    
45	. C . . . . . C C C .    
46	. C . . . . . C C C .    
47	. A . . . . . A A A .    
48	. T . . . . . T T T .    
49	. T . . . . . T T T .    
50	. T . . . . . T T T .    
51	. T . . . . . T T T .    
52	. G . . . . . G G G .    
53	. C . . . C . C C C .    
54	. G . . . G . G G G .    
55	. T . . . T T T T T .    
56	. T . . . T T T T T .    
57	. A . . . A A A A A .    
58	. C . . . C C C C C C    
59	. C . . . C C C C C C    
60	. A . . . A A A A A A    
61	. C . C . C C C C C C    
62	. A . A . A A A A A A    
63	. G . G . G G G G G G    
64	. C . C . C C C C C C    
65	. G . G . G G G G G G    
66	. A . A . A A A A A A    
67	. G . G . G G G G G G    
68	. G . G . G G G G G G    
69	. G . G . G G G G G G    
70	. G . G . G G G G G G    
71	. G . G . G G G G G G    
72	. A . A . A A A A A A    
73	. C . C . C C C C C C    
74	T T . T T T T T T T T    
75	T T . T T T T T T T T    
76	T T . T T T T T T T T    
77	G G . G G G G G G G G    
78	G G . G G G G G G G G    
79	C C . C C C C C C C C    
80	A A . A A A A A A A A    
81	T T . T T T T T T T T    
82	T T . T T T T T T T T    
83	T T . T T T T T T T T    
84	T T . T T T T T T T T    
85	A A . A A A A A A A A    
86	A A . A A A A A A A A    
87	T T . T T T T T T T T    
88	C C . C C C C C C C C    
89	T T . T T T T T T T T    
90	A A . A A A A A A A A    
91	T T . T T T T T T T T    
92	T T . T A T T A T T T    
93	A A . A A A A A A A A    
94	A A . A A A A A A A A    
95	T T . T T T T T T T T    
96	T T . T T T T T T T T    
97	T T . T T T T T T T T    
98	T T . T T T T T T T T    
99	T T . T T T T T T T T    
100	T T . T T T T T T T T    
101	G G . G G G G G G G G    
102	A A . A A A A A A A A    
103	C C . C C C C C C C C    
104	G G . G G G G G G G G    
105	T T . T T T T T T T T    
106	A A . A A A A A A A A    
107	A A . A A A A A A A A    
108	G G . G G G G G G G G    
109	A A . A A A A A A A A    
110	C C . C C C C C C C C    
111	G G . G G G G G G G G    
112	C C . C C C C C C C C    
113	A A . A A A A A A A A    
114	T T . T T T T T T T T    
115	C C . C C C C C C C C    
116	A A . A A A A A A A A    
117	T T . T T T T T T T T    
118	T T . T T T T T T T T    
119	G G . G G G G G G G G    
120	T T . T T T T T T T T    
121	A A . A A A A A A A A    
122	A A . A A A A A A A A    
123	C C . C C C C C C C C    
124	G G . G G G G G G G G    
125	T T . T T T T T T T T    
126	T T . T T T T T T T T    
127	A A . A A A A A A A A    
128	A A . A A A A A A A A    
129	G G . G G G G G G G G    
130	T T . T T T T T T T T    
131	G G . G G G G G G G G    
132	C C . C C C C C C C C    
133	A A . A A A A A A A A    
134	T T . T T T T T T T T    
135	T T . T T T T T T T T    
136	G G . G G G G G G G G    
137	A A . A A A A A A A A    
138	C C . C C C C C C C C    
139	T T . T T T T T T T T    
140	T T . T T T T T T T T    
141	T T . T T T T T T T T    
142	T T . T T T T T T T T    
143	T T . T T T T T T T T    
144	A A . A A A A A A A A    
145	T T . T T T T T T T T    
146	A A . A A A A A A A A    
147	C C . C C C C C C C C    
148	T T . T T T T T T T T    
149	C C . C C C C C C C C    
150	G G . G G G G G G G G    
151	A A . A A A A A A A A    
152	A A . A A A A A A A A    
153	C C . C C C C C C C C    
154	A A . A A A A A A A A    
155	T T . T T T T T T T T    
156	T T . T T T T T T T T    
157	T T . T T T T T T T T    
158	T T . T T T T T T T T    
159	A A . A A A A A A A A    
160	T T . T T T T T T T T    
161	T T . T T T T T T T T    
162	T T . T T T T T T T T    
163	T T . T T T T T T T T    
164	G G . G G G G G G G G    
165	A A . A A A A A A A A    
166	T T . T T T T T T T T    
167	T T . T T T T T T T T    
168	C C . C C C C C C C C    
169	A A . A A A A A A A A    
170	G G . G G G G G G G G    
171	T T . T T T T T T T T    
172	T T . T T T T T T T T    
173	G G . G G G G G G G G    
174	A A . A A A A A A A A    
175	A A . A A A A A A A A    
176	T T . T T T T T T T T    
177	T T . T T T T T T T T    
178	T T . T T T T T T T T    
179	A A . A A A A A A A A    
180	T T . T T T T T T T T    
181	C C . C C C C C C C C    
182	C C . C C C C C C C C    
183	A A . A A A A A A A A    
184	G G . G G G G G G G G    
185	C C . C C C C C C C C    
186	T T . T T T T T T T T    
187	G G . G G G G G G G G    
188	T T . T T T T T T T T    
189	A A . A A A A A A A A    
190	T T . T T T T T T T T    
191	A A . A A A A A A A A    
192	A A . A A A A A A A A    
193	T T . T T T T T T T T    
194	T T . T T T T T T T T    
195	G G . G G G G G G G G    
196	T T . T T T T T T T T    
197	G G . G G G G G G G G    
198	A A . A A A A A A A A    
199	A A . A A A A A A A A    
200	T T . T T T T T T T T    
201	G G . G G G G G G G G    
202	A A . A A A A A A A A    
203	T T . T T T T T T T T    
204	C C . C C C C C C C C    
205	T T . T T T T T T T T    
206	A A . A A A A A A A A    
207	G G . G G G G G G G G    
208	C C . C C C C C C C C    
209	T T . T T T T T T T T    
210	A A . A A A A A A A A    
211	A A . A A A A A A A A    
212	C C . C C C C C C C C    
213	T T . T T T T T T T T    
214	T T . T T T T T T T T    
215	A A . A A A A A A A A    
216	G G . G G G G G G G G    
217	C C . C C C C C C C C    
218	T T . T T T T T T T T    
219	G G . G G G G G G G G    
220	G G . G G G G G G G G    
221	C C . C C C C C C C C    
222	T T . T T T T T T T T    
223	G G . G G G G G G G G    
224	A A . A A A A A A A A    
225	G G . G G G G G G G G    
226	T T . T T T T T T T T    
227	T T . T T T T T T T T    
228	G G . G G G G G G G G    
229	A A . A A A A A A A A    
230	T T . T T T T T T T G    
231	A A . A A A A A A A A    
232	T T . T T T T T T T T    
233	T T . T T T T T T T T    
234	A A . A A A A A A A A    
235	C C . C C C C C C C C    
236	A A . A A A A A A A A    
237	A A . A A A A A A A A    
238	A A . A A A A A A A A    
239	C C . C C C C C C C C    
240	G G . G G G G G G G G    
241	G G . G G G G G G G G    
242	C C . C C C C C C C C    
243	T T . T T T T T T T T    
244	A A . A A A A A A A A    
245	A A . A A A A A A A A    
246	C C . C C C C C C C C    
247	T T . T T T T T T T T    
248	C C . C C C C C C C C    
249	A A . A A A A A A A A    
250	C C . C C C C C C C C    
251	A A . A A A A A A A A    
252	G G . G G G G G G G G    
253	A A . A A A A A A A A    
254	C C . C C C C C C C C    
255	A A . A A A A A A A A    
256	C C . C C C C C C C C    
257	G G . G G G G G G G G    
258	A A . A A A A A A A A    
259	T T . T T T T T T T T    
260	T T . T T T T T T T T    
261	T T . T T T T T T T T    
262	A A . A A A A A A A A    
263	T T . T T T T T T T T    
264	T T . T T T T T T T T    
265	T T . T T T T T T T T    
266	T T . T T T T T T T T    
267	T T . T T T T T T T T    
268	T T . T T T T T T T T    
269	A A . A A A A A A A A    
270	A A . A A A A A A A A    
271	A A . A A A A A A A A    
272	C C . C C C C C C C C    
273	A A . A A A A A A A A    
274	T T . T T T T T T T T    
275	- - . - T T T T - - T  5	 indel  
276	- - . - T T T T - - T  5	 indel  
277	T T . T T T T T T T T    
278	G G . G G G G G G G G    
279	T T . T T T T T T T T    
280	G G . G G G G G G G G    
281	A A . A A A A A A A A    
282	A A . A A A A A A A A    
283	A A . A A A A A A A A    
284	T T . T T T T T T T T    
285	G G . G G G G G G G G    
286	T T . T T T T T T T T    
287	T T . T T T T T T T T    
288	C C . C C C C C C C C    
289	T T . T T T T T T T T    
290	T T . T T T T T T T T    
291	G G . G G G G G G G G    
292	G G . G G G G G G G G    
293	A A . A A A A A A A A    
294	C C . C C C C C C C C    
295	A A . A A A A A A A A    
296	G G . G G G G G G G G    
297	C C . C C C C C C C C    
298	A A . A A A A A A A A    
299	T T . T T T T T T T T    
300	G G . G G G G G G G G    
301	G G . G G G G G G G G    
302	A A . A A A A A A A A    
303	A A . A A A A A A A A    
304	A A . A A A A A A A A    
305	C C . C C C C C C C C    
306	T T . T T T T T T T T    
307	A A . A A A A A A A A    
308	A A . A A A A A A A A    
309	A A . A A A A A A A A    
310	G G . G G G G G G G G    
311	T T . T T T T T T T T    
312	G G . G G G G G G G G    
313	G G . G G G G G G G G    
314	A A . A A A A A A A A    
315	A A . A A A A A A A A    
316	T T . T T T T T T T T    
317	T T . T T T T T T T T    
318	C C . C C C C C C C C    
319	G G . G G G G G G G G    
320	A A . A A A A A A A A    
321	A A . A A A A A A A A    
322	G G . G G G G G G G G    
323	T T . T T T T T T T T    
324	A A . A A A A A A A A    
325	C C . C C C C C C C C    
326	T T . T T T T T T T T    
327	G G . G G G G G G G G    
328	A A . A A A A A A A A    
329	A A . A A A A A A A A    
330	A A . A A A A A A A A    
331	C C . C C C C C C C C    
332	C C . C C C C C C C C    
333	T T . T T T T T T T T    
334	T T . T T T T T T T T    
335	G G . G G G G G G G G    
336	C C . C C C C C C C C    
337	G G . G G G G G G G G    
338	A A . A A A A A A A A    
339	C C . C C C C C C C C    
340	T T . T T T T T T T T    
341	C C . C C C C C C C C    
342	C C . C C C C C C C C    
343	A A . A A A A A A A A    
344	G G . G G G G G G G G    
345	A A . A A A A A A A A    
346	A A . A A A A A A A A    
347	G G . G G G G G G G G    
348	A A . A A A A A A A A    
349	T T . T T T T T T T T    
350	C C . C C C C C C C C    
351	T T . T T T T T T T T    
352	G G . G G G G G G G G    
353	G G . G G G G G G G G    
354	T T . T T T T T T T T    
355	A A . A A A A A A A A    
356	A A . A A A A A A A A    
357	A A . A A A A A A A A    
358	C C . C C C C C C C C    
359	T T . T T T T T T T T    
360	T T . T T T T T T T T    
361	T T . T T T T T T T T    
362	C C . C C C C C C C C    
363	A A . A A A A A A A A    
364	A A . A A A A A A A A    
365	A A . A A A A A A A A    
366	G G . G G G G G G G G    
367	T T . T T T T T T T T    
368	G G . G G G G G G G G    
369	C C . C C C C C C C C    
370	A A . A A A A A A A A    
371	G G . G G G G G G G G    
372	A A . A A A A A A A A    
373	C C . C C C C C C C C    
374	T T . T T T T T T T T    
375	G G . G G G G G G G G    
376	A A . A A A A A A A A    
377	G G G G G G G G G G G    
378	G G G G G G G G G G G    
379	A A A A A A A A A A A    
380	A A A A A A A A A A A    
381	A A A A A A A A A A A    
382	C C C C C C C C C C C    
383	T T T T T T T T T T T    
384	T T T T T T T T T T T    
385	C C C C C C C C C C C    
386	T T T T T T T T T T T    
387	C C C C C C C C C C C    
388	C C C C C C C C C C C    
389	A A A A A A A A A A A    
390	T T T T T T T T T T T    
391	T T T T T T T T T T T    
392	T T T T T T T T T T T    
393	G G G G G G G G G G G    
394	A A A A A A A A A A A    
395	G G G G G G G G G G G    
396	T T T T T T T T T T T    
397	T T T T T T T T T T T    
398	C C C C C C C C C C C    
399	C C C C C C C C C C C    
400	A A A A A A A A A A A    
401	A A A A A A A A A A A    
402	G G G G G G G G G G G    
403	G G G G G G G G G G G    
404	A A A A A A A A A A A    
405	A A A A A A A A A A A    
406	G G G G G G G G G G G    
407	G G G G G G G G G G G    
408	C C C C C C C C C C C    
409	C C C C C C C C C C C    
410	T T T T T T T T T T T    
411	G G G G G G G G G G G    
412	A A A A A A A A A A A    
413	A A A A A A A A A A A    
414	A A A A A A A A A A A    
415	C C C C C C C C C C C    
416	G G G G G G G G G G G    
417	G G G G G G G G G G G    
418	T T T T T T T T T T T    
419	G G G G G G G G G G G    
420	G G G G G G G G G G G    
421	C C C C C C C C C C C    
422	G G G G G G G G G G G    
423	C C C C C C C C C C C    
424	A A A A A A A A A A A    
425	C C C C C C C C C C C    
426	C C C C C C C C C C C    
427	A A A A A A A A A A A    
428	A A A A A A A A A A A    
429	C C C C C C C C C C C    
430	C C C C C C C C C C C    
431	T T T T T T T T T T T    
432	T T T T T T T T T T T    
433	G G G G G G G G G G G    
434	T T T T T T T T T T T    
435	C C C C C C C C C C C    
436	A A A A A A A A A A A    
437	A A A A A A A A A A A    
438	A A A A A A A A A A A    
439	G G G G G G G G G G G    
440	C C C C C C C C C C C    
441	C C C C C C C C C C C    
442	A A A A A A A A A A A    
443	C C C C C C C C C C C    
444	T T T T T T T T T T T    
445	C C C C C C C C C C C    
446	T T T T T T T T T T T    
447	C C C C C C C C C C C    
448	A A A A A A A A A A A    
449	A A A A A A A A A A A    
450	A A A A A A A A A A A    
451	A A A A A A A A A A A    
452	A A A A A A A A A A A    
453	A A A A A A A A A A A    
454	- - - - - - - - - - A    
455	T T T T T T T T T T T    
456	A A A A A A A A A A A    
457	C C C C C C C C C C C    
458	A A A A A A A A A A A    
459	T T T T T T T T C T T    
460	T T T T T T T T T T T    
461	C C C C C C C C C C C    
462	G G G G G G G G G G G    
463	T T T T T T T T T T T    
464	T T T T T T T T T T T    
465	G G G G G G G G G G G    
466	C C C C C C C C C C C    
467	G G G G G G G G G G G    
468	A A A A A A A A A A A    
469	A A A A A A A A A A A    
470	C C C C C C C C C C C    
471	A A A A A A A A A A A    
472	G G G G G G G G G G G    
473	A A A A A A A A A A A    
474	A A A A A A A A A A A    
475	G G G G G G G G G G G    
476	A A A A A A A A A A A    
477	G G G G G G G G G G G    
478	G G G G G G G G G G G    
479	G G G G G G G G G G G    
480	T T T T T T T T T T T    
481	G G G G G G G G G G G    
482	C C C C C C C C C C C    
483	T T T T T T T T T T T    
484	C C C C C C C C C C C    
485	A A A A A A A A A A A    
486	C C C C C C C C C C C    
487	T T T T T T T T T T T    
488	A A A A A A A A A A A    
489	A A A A A A A A A A A    
490	A A A A A A A A A A A    
491	. C C C C C C C C C C    
492	. C C C C C C C C C C    
493	. G G G G G G G G G G    
494	. G G G G G G G G G G    
495	. T T T T T T T T T T    
496	. C C C C C C C C C C    
497	. A A A A A A A A A A    
498	. A A A A A A A A A A    
499	. C C C C C C C C C C    
500	. C C C C C C C C C C    
501	. A A A A A A A A A A    
502	. A A A A A A A A A A    
503	. G G G G G G G G G G    
504	. A A A A A A A A A A    
505	. A A A A A A A A A A    
506	. C C C C C C C C C C    
507	. A A A A A A A A A A    
508	. A A A A A A A A A A    
509	. C C C C C C C C C C    
510	. T T T T T T T T T T    
511	. T T T T T T T T T T    
512	. T T T T T T T T T T    
513	. T T T T T T T T T T    
514	. G G G G G G G G G G    
515	. A A A A A A A A A A    
516	. C C C C C C C C C C    
517	. G G G G G G G G G G    
518	. G G G G G G G G G G    
519	. G G G G G G G G G G    
520	. C C C C C C C C C C    
521	. A A A A A A A A A A    
522	. A A A A A A A A A A    
523	. C C C C C C C C C C    
524	. G G G G G G G G G G    
525	. G G G G G G G G G G    
526	. C C C C C C C C C C    
527	. G G G G G G G G G G    
528	. A A A A A A A A A A    
529	. A A A A A A A A A A    
530	. G G G G G G G G G G    
531	. A A A A A A A A A A    
532	. T T T T T T T T T T    
533	. A A A A A A A A A A    
534	. T T T T T T T T T T    
535	. G G G G G G G G G G    
536	. A A A A A A A A A A    
537	. C C C C C C C C C C    
538	. G G G G G G G G G G    
539	. T T T T T T T T T T    
540	. A A A A A A A A A A    
541	. T T T T T T T T T T    
542	. A A A A A A A A A A    
543	. T T T T T T T T T T    
544	. G G G G G G G G G G    
545	. G G G G G G G G G G    
546	. C C C C C C C C C C    
547	. G G G G G G G G G G    
548	. A A A A A A A A A A    
549	. C C C C C C C C C C    
550	. C C C C C C C C C C    
551	. G G G G G G G G G G    
552	. A A A A A A A A A A    
553	. G G G G G G G G G G    
554	. A A A A A A A A A A    
555	. A A A A A A A A A A    
556	. T T T T T T T T T T    
557	. A A A A A A A A A A    
558	. T T T T T T T T T T    
559	. C C C C C C C C C C    
560	. G G G G G G G G G G    
561	. G G G G G G G G G G    
562	. C C C C C C C C C C    
563	. A A A A A A A A A A    
564	. G G G G G G G G G G    
565	. A A A A A A A A A A    
566	. A A A A A A A A A A    
567	. T T T T T T T T T T    
568	. T T T T T T T T T T    
569	. A A A A A A A A A A    
570	. C C C C C C C C C C    
571	. A A A A A A A A A A    
572	. C C C C C C C C C C    
573	. G G G G G G G G G G    
574	. A A A A A A A A A A    
575	. C C A C C C C C C C    
576	. C C C C C C C C C C    
577	. A A A A A A A A A A    
578	. C C C C C C C C C C    
579	. T T T T T T T T T T    
580	. A A A A A A A A A A    
581	. G G G G G G G G G G    
582	. G G G G G G G G G G    
583	. C C C C C C C C C C    
584	. G G G G G G G G G G    
585	. C C C C C C C C C C    
586	. A A A A A A A A A A    
587	. A A A A A A A A A A    
588	. T T T T T T T T T T    
589	. G G G G G G G G G G    
590	. G G G G G G G G G G    
591	. C C C C C C C C C C    
592	. G G G G G G G G G G    
593	. C C C C C C C C C C    
594	. C C C C C C C C C C    
595	. A A A A A A A A A A    
596	. A A A A A A A A A A    
597	. A A A A A A A A A A    
598	. G G G G G G G G G G    
599	. A A A A A A A A A A    
600	. A A A A A A A A A A    
601	. T T T T T T T T T T    
602	. A A A A A A A A A A    
603	. C C C C C C C C C C    
604	. A A A A A A A A A A    
605	. A A A A A A A A A A    
606	. C C C C C C C C C C    
607	. A A A A A A A A A A    
608	. C C C C C C C C C C    
609	. A A A A A A A A A A    
610	. G G G G G G G G G G    
611	. T T T T T T T T T T    
612	. A A A A A A A A A A    
613	. T T T T T T T T T T    
614	. C C C C C C C C C C    
615	. T T T T T T T T T T    
616	. G G G G G G G G G G    
617	. A A A A A A A A A A    
618	. T T T T T T T T T T    
619	. G G G G G G G G G G    
620	. G G G G G G G G G G    
621	. A A A A A A A A A A    
622	. C C C C C C C C C C    
623	. A A A A A A A A A A    
624	. T T T T T T T T T T    
625	. . T T T T T T T T T    
626	. . G G G G G G G G G    
627	. . T T T T T T T T T    
628	. . A A A A A A A A A    
629	. . T T T T T T T T T    
630	. . A A A A A A A A A    
631	. . C C C C C C C C C    
632	. . G G G G G G G G G    
633	. . A A A A A A A A A    
634	. . C C C C C C C C C    
635	. . G G G G G G G G G    
636	. . A A A A A A A A A    
637	. . C C C C C C C C C    
638	. . A A A A A A A A A    
639	. . T T T T T T T T T    
640	. . G G G G G G G G G    
641	. . C C C C C C C C C    
642	. . A A A A A A A A A    
643	. . C C C C C C C C C    
644	. . A A A A A A A A A    
645	. . T T T T T T T T T    
646	. . T T T T T T T T T    
647	. . G G G G G G G G G    
648	. . A A A A A A A A A    
649	. . C C C C C C C C C    
650	. . A A A A A A A A A    
651	. . C C C C C C C C C    
652	. . C C C C C C C C C    
653	. . A A A A A A A A A    
654	. . G G G G G G G G G    
655	. . T T T T T T T T T    
656	. . T T T T T T T T T    
657	. . C C C C C C C C C    
658	. . C C C C C C C C C    
659	. . A A A A A A A A A    
660	. . A A A A A A A A A    
661	. . C C C C C C C C C    
662	. . C C C C C C C C C    
663	. . A A A A A A A A A    
664	. . G G G G G G G G G    
665	. . A T T T T T T T T    
666	. . . T T T T T T T T    
667	. . . C C C C C C C C    
668	. . . A A A A A A A A    
669	. . . A A A A A A A A    
670	. . . G G G G G G G G    
671	. . . T T T T T T T T    
672	. . . C C C C C C C C    
673	. . . G G G G G G G G    
674	. . . C C C C C C C C    
675	. . . A A A A A A A A    
676	. . . A A A A A A A A    
677	. . . C C C C C C C C    
678	. . . C C C C C C C C    
679	. . . A A A A A A A A    
680	. . . G G G G G G G G    
681	. . . T T T T T T T T    
682	. . . T T T T T T T T    
683	. . . T T T T T T T T    
684	. . . C C C C C C C C    
685	. . . A A A A A A A A    
686	. . . T T T T T T T T    
687	. . . . T T T T T T T    
688	. . . . T T T T T T T    
689	. . . . G G G G G G G    
690	. . . . T T T T T T T    
691	. . . . T T T T T T T    
692	. . . . T T T T T T T    
693	. . . . C C C C C C C    
694	. . . . T T T T T T T    
695	. . . . A A A A A A A    
696	. . . . G G G G G G G    
697	. . . . C C C C C C C    
698	. . . . G G G G G G G    
699	. . . . C C C C C C C    
700	. . . . C C C C C C C    
701	. . . . T T T T T T T    
702	. . . . C C C C C C C    
703	. . . . T T T T T T T    
704	. . . . C C C C C C C    
705	. . . . T T T T T T T    
706	. . . . T T T T T T T    
707	. . . . T T T T T T T    
708	. . . . C C C C C C C    
709	. . . . A A A A A A A    
710	. . . . C C C C C C C    
711	. . . . C C C C C C C    
712	. . . . C C C C C C C    
713	. . . . A A A A A A A    
714	. . . . G G G G G G G    
715	. . . . G G G G G G G    
716	. . . . A A A A A A A    
717	. . . . G G G G G G G    
718	. . . . C C C C C C C    
719	. . . . G G G G G G G    
720	. . . . C C C C C C C    
721	. . . . G G G G G G G    
722	. . . . T T T T T T T    
723	. . . . G G G G G G G    
724	. . . . T T T T T T T    
725	. . . . G G G G G G G    
726	. . . . A A A A A A A    
727	. . . . A A A A A A A    
728	. . . . A A A A A A A    
729	. . . . G G G G G G G    
730	. . . . T T T T T T T    
731	. . . . T T T T T T T    
732	. . . . C C C C C C C    
733	. . . . T T T T T T T    
734	. . . . C C C C C C C    
735	. . . . T T T T T T T    
736	. . . . T T T T T T T    
737	. . . . G G G G G G G    
738	. . . . A A A A A A A    
739	. . . . T T T T T T T    
740	. . . . T T T T T T T    
741	. . . . T T T T T T T    
742	. . . . T . T T T T T    
743	. . . . C . G C C C C    
744	. . . . A . . A A A A    
745	. . . . . . . A A A A    
746	. . . . . . . C C C C    
747	. . . . . . . A A A A    
748	. . . . . . . G G G G    
749	. . . . . . . A A A A    
750	. . . . . . . G G G G    
751	. . . . . . . G G G G    
752	. . . . . . . G G G G    
753	. . . . . . . A A A A    
754	. . . . . . . C C C C    
755	. . . . . . . T T T T    
756	. . . . . . . T T T T    
757	. . . . . . . C C C C    
758	. . . . . . . G G G G    
759	. . . . . . . A A A A    
760	. . . . . . . C C C C    
761	. . . . . . . G G G G    
762	. . . . . . . A A A A    
763	. . . . . . . A A A A    
764	. . . . . . . A A A A    
765	. . . . . . . T T T T    
766	. . . . . . . G G G G    
767	. . . . . . . T T T T    
768	. . . . . . . A A A A    
769	. . . . . . . T T T T    
770	. . . . . . . C C C C    
771	. . . . . . . T T T T    
772	. . . . . . . C C C C    
773	. . . . . . . C C C C    
774	. . . . . . . A A A A    
775	. . . . . . . T T T T    
776	. . . . . . . T T T T    
777	. . . . . . . T T T T    
778	. . . . . . . T T T T    
779	. . . . . . . G G G G    
780	. . . . . . . G G G G    
781	. . . . . . . A A A A    
782	. . . . . . . T T T T    
783	. . . . . . . C C C C    
784	. . . . . . . A A A A    
785	. . . . . . . T T T T    
786	. . . . . . . G G G G    
787	. . . . . . . T T T T    
788	. . . . . . . T T T T    
789	. . . . . . . G G G G    
790	. . . . . . . A A A A    
791	. . . . . . . T T T T    
792	. . . . . . . C C C C    
793	. . . . . . . A A A A    
794	. . . . . . . G G G G    
795	. . . . . . . G G G G    
796	. . . . . . . C C C C    
797	. . . . . . . T T T T    
798	. . . . . . . T T T T    
799	. . . . . . . G G G G    
800	. . . . . . . A A A A    
801	. . . . . . . A A A A    
802	. . . . . . . C C C C    
803	. . . . . . . C C C C    
804	. . . . . . . A A A A    
805	. . . . . . . G G G G    
806	. . . . . . . T T T T    
807	. . . . . . . C C C C    
808	. . . . . . . T T T T    
809	. . . . . . . G G G G    
810	. . . . . . . G T T T    
811	. . . . . . . . G G G    
812	. . . . . . . . G G G    
813	. . . . . . . . C C C    
814	. . . . . . . . A A A    
815	. . . . . . . . G G G    
816	. . . . . . . . A A A    
817	. . . . . . . . G G G    
818	. . . . . . . . C C C    
819	. . . . . . . . T T T    
820	. . . . . . . . G G G    
821	. . . . . . . . A A A    
822	. . . . . . . . C C C    
823	. . . . . . . . C C C    
824	. . . . . . . . A A A    
825	. . . . . . . . A A A    
826	. . . . . . . . T T T    
827	. . . . . . . . G G G    
828	. . . . . . . . C C C    
829	. . . . . . . . A A A    
830	. . . . . . . . T T T    
831	. . . . . . . . T T T    
832	. . . . . . . . G G G    
833	. . . . . . . . A A A    
834	. . . . . . . . C C C    
835	. . . . . . . . T T T    
836	. . . . . . . . C C C    
837	. . . . . . . . T T T    
838	. . . . . . . . A A A    
839	. . . . . . . . A A A    
840	. . . . . . . . A A A    
841	. . . . . . . . C C C    
842	. . . . . . . . C C C    
843	. . . . . . . . T T T    
844	. . . . . . . . C C C    
845	. . . . . . . . G G G    
846	. . . . . . . . C C C    
847	. . . . . . . . A A A    
848	. . . . . . . . A A A    
849	. . . . . . . . A A A    
850	. . . . . . . . C C C    
851	. . . . . . . . C C C    
852	. . . . . . . . A A A    
853	. . . . . . . . A A A    
854	. . . . . . . . G G G    
855	. . . . . . . . C C C    
856	. . . . . . . . G G G    
857	. . . . . . . . A A A    
858	. . . . . . . . C C C    
859	. . . . . . . . G G G    
860	. . . . . . . . C C C    
861	. . . . . . . . A A A    
862	. . . . . . . . C C C    
863	. . . . . . . . C C C    
864	. . . . . . . . G G G    
865	. . . . . . . . G G G    
866	. . . . . . . . A . A    
867	. . . . . . . . C . C    
868	. . . . . . . . G . G    
869	. . . . . . . . C . C    
870	. . . . . . . . T . T    
871	. . . . . . . . G . G    
872	. . . . . . . . C . C    
873	. . . . . . . . C . C    
874	. . . . . . . . T . T    
875	. . . . . . . . G . G    
876	. . . . . . . . . . T    
877	. . . . . . . . . . C    
878	. . . . . . . . . . G    
879	. . . . . . . . . . G    
880	. . . . . . . . . . T    
881	. . . . . . . . . . G    
882	. . . . . . . . . . G    
883	. . . . . . . . . . G    
884	. . . . . . . . . . C    
885	. . . . . . . . . . G    
886	. . . . . . . . . . C    
887	. . . . . . . . . . T    
888	. . . . . . . . . . C    
889	. . . . . . . . . . G    
890	. . . . . . . . . . C    
891	. . . . . . . . . . T    
892	. . . . . . . . . . C    
893	. . . . . . . . . . G    
894	. . . . . . . . . . C    
895	. . . . . . . . . . A    
896	. . . . . . . . . . G    
897	. . . . . . . . . . C    
898	. . . . . . . . . . G    
899	. . . . . . . . . . T    
900	. . . . . . . . . . C    
901	. . . . . . . . . . G    
902	. . . . . . . . . . C
```
